# Prevalence of pancreatic autoantibodies in non-diabetic patients with autoimmune thyroid disease and its relation to insulin secretion and glucose tolerance

**DOI:** 10.1590/2359-3997000000280

**Published:** 2017-06-23

**Authors:** Carolina Sallorenzo, Regina Silva, Teresa Kasamatsu, Sérgio Dib

**Affiliations:** 1 Departamento de Medicina Universidade Federal de São Paulo São Paulo SP Brasil Divisão de Endocrinologia, Departamento de Medicina, Universidade Federal de São Paulo (Unifesp), São Paulo, SP, Brasil

**Keywords:** Autoimmune thyroid disease, pancreatic autoantibodies, glucose tolerance, beta-cell function

## Abstract

**Objective:**

We evaluated the prevalence of glutamic acid decarboxylase (GADA) and tyrosine phosphatase-protein antibodies (IA2A), their titers and their relation to first phase insulin response (FPIR) and glucose tolerance in autoimmune thyroid diseases (ATDs) patients.

**Subjects and methods:**

Graves’ disease (GD; n = 181) and Hashimoto’s thyroiditis (HT; n = 143) patients in addition to healthy controls (n = 93) were studied. Secondly, FPIR and oral glucose tolerance tests (OGTT) were performed in 11 anti-pancreatic islet–cell (+) and in 20 anti-pancreatic–cell (-) patients.

**Results:**

There was a non significant trend for higher prevalence of GADA positivity in GD vs HT (7.2% vs 2% p = 0.06), but the GADA titers were higher in HT. We also did not find a significant difference in IA2 prevalence (0.7% vs 0.0%) between these two groups or compared to the control group. In the subsequent analysis, low FPIR was found in 10% of these patients but without statistical difference for OGTT between pancreatic antibody–positive and –negative patients.

**Conclusion:**

A trend for greater prevalence of GADA was observed for GD patients than for HT or control. However, the titers of these autoantibodies were higher in HT patients, but there was no significant relation to insulin secretion and glucose tolerance at that moment and stage of autoimmune diseases.

## INTRODUCTION

Autoimmune thyroid diseases (ATDs), represented by Graves’ disease (GD) and Hashimoto’s thyroiditis (HT) are very common, affecting up to 9% of population (1). It has been accepted that endocrine autoimmune diseases coexist in the same person and even in the same families. This association can be explained, in part, by a shared genetic background and is mainly linked to the HLA system and the CTLA-4, CD25, PTPN22 and FOXP3 genes (2).

The increased risk of an additional autoimmune disease in a subject with a primary diagnosis of ATD can be quantified (3).

Risk assessment of developing type 1A diabetes (T1AD) in adults can begin with the evaluation of genetics and autoantibodies to glutamic acid decarboxylase (GADA) and the protein tyrosine phosphatase antigen IA-2 (IA-2A), among other pancreatic autoantibody sera markers for T1AD (4,5). The risk of clinical disease is related to the titers (6,7) and the number of additional autoantibodies (8). However, the decreasing rate of beta-cell function is not homogeneous, and the subjects at risk for T1AD can express these autoantibodies for years prior to disease diagnosis (8). The metabolic abnormalities of the pre-diabetic period are not well understood. The presence of multiple pancreatic autoantibodies and impaired first phase insulin response (FPIR) are known to be strong risk factors to T1AD clinical development (9). Initial abnormalities in oral glucose tolerance tests (OGTTs) are frequently silent and can precede the diagnosis of T1AD (10). Both OGTT and intravenous glucose tolerance test (IVGTT) may also be modulated by insulin resistance (11).

The autoimmune process against islet beta-cells in adults is generally slow and can take years before clinical T1AD (12-15) appears. Also, there are reports on GADA patterns in autoimmune polyendocrine syndrome that can be different from those in sporadic T1AD patients. GADA has been detected in 6-13% of GD and in 3-8% of HT patients (16-18). In a previous study, we evaluated the frequency of GADA in GD, HT and T1AD Brazilian patients (19) and found that despite the genetic diversity of our population, the frequency of GADA in Brazilians with these diseases is similar to their pairs from other countries.

Few studies have addressed the relation between islet-cell autoimmunity and glucose homeostasis in non-diabetic ATD Brazilian patients. Here, we evaluated the prevalence of GADA and IA2A and their relation to first-phase insulin secretion and glucose tolerance in non-diabetic individuals with ATDs.

## SUBJECTS AND METHODS

Patients were recruited between March 2001 and July 2003. The study cohort (n = 324) was randomized from a southeast Brazilian population attending the outpatient endocrine clinic of a university hospital for follow-up of their ATDs. All were non-diabetic patients. In addition, as a control group, 93 blood donors, healthy subjects without T1AD, ATD or neither autoimmune disease’s first-degree relatives were studied.

All subjects gave informed written consent to participate. The study was approved by the Ethics Committee on Human Research of the University Hospital of São Paulo Federal University. GD was defined by the presence of biochemical hyperthyroidism with the following: I) diffuse uptake on a radionuclide scan/diffuse goiter on ultrasound scan; II) Graves’ ophthalmopathy; III) positive autoantibodies to the thyroid-stimulating receptor or IV) diffuse goiter on physical examination HT diagnosed by documented clinical and biochemical hypothyroidism requiring L-thyroxine replacement therapy and the presence of antithyroperoxidase (TPOAb) and antithyroglobulin antibodies (TgAb) and/or histologically by fine-needle biopsy of the thyroid. All patients were treated for thyroid disease and taking the following medicine: levothyroxine sodium, methimazole and radioiodine (I131) therapy.

Eleven euthyroid positive and twenty negative GADA and/or IA2A (a subgroup from the main ATD patient group) with a similar age, gender and BMI were submitted to an OGTT. All but one GADA/IA2Ab+ patient (due to technical problems) were submitted to an IVGTT (20) to evaluate the FPIR.

Baseline blood samples were obtained for measurements of glucose, insulin, thyroid hormones and autoantibodies.

Glucose was measured using the glucose hexokinase II enzymatic method.

Serum insulin was measured using a time-resolved fluoroimmunoassay (AutoDelfia Insulin-Perkin Elmer Life Sciences, Finland) with a reference range: 14.04–158.4 pmol/L; the intra- and inter-assay coefficients of variation (CV) varied from 1.7% (serum insulin: 180.6 pmol/L) to 2.4% (serum insulin: 33.0 pmol/L) and 3.5% to 2.3%, respectively.

TPOAb and TgAb were measured in our laboratory using highly purified recombinant human TPO antigen (Fitzgerald Industries International, Inc, Concord, USA) and a human-purified Tg antigen employing flow cytometry (Luminex) (21). The cut-off limit was 14 and 26 UI/ml for TPOAb and TgAb, respectively. TPOAb intra-assay CVs were 14.3% and 12.6% to mean values of 2.3 ± 0.33 and 621 ± 78 UI/ml, respectively, and inter-assay CVs were 33% and 20% to mean values of 1.8 ± 0.6 and 688 ± 139 UI/ml, respectively. Tg Ab intra-assay CVs were 9.2% and 9.6% to mean values of 2.4 ± 0.22 and 811 ± 78 UI/ml, respectively, and inter-assays of 36% and 19.6% to mean values of 2.3 ± 0.86 and 905 ± 177 UI/ml, respectively.

TSH receptor antibodies (TRAb) were determined using a radioreceptor assay with reagents provided by BRAHMS AG (Henninsdorf, Germany). The normal value was < 10%, and the intra and inter-assay CVs were < 10% and 13.4%, respectively.

Serum TSH was measured using an immunofluorimetric assay developed in our laboratory (reference ranges: 0.3–5.0 mUI/L; intra- and inter-assay CVs of 9.5% and 11.6%, respectively).

GADA 65 and IA2A were measured from frozen serum samples using a commercial radioimmunoassay kit RSR (UK, England). The results were expressed in U/ml. The cut-off limit for GADA was 1.72 U/ml and was 0.97U/ml for IA2A, which were estimated to be in the 99^th^ percentile of the results obtained when we analyzed the sera from 194 healthy controls in our laboratory (22). The GADA intra and inter-assay CV were 3.58% and 4.9%, respectively. The IA2A intra- and inter-assay CVs were 4.3% and 3.4%, respectively.

The OGTT was performed according to the American Diabetes Association’s standards (23). IVGTT was performed in patients as previously described, after an overnight fast of 10 hours, between 7:00–9:00 am, and blood samples were drawn for measurement of glucose and insulin 5 minutes before and at time 0 and 1, 3, 5, 10 minutes after an IV infusion of 25% glucose (0.5 g/kg of body weight up to 35 g) (20). FPIR was determined as the sum of 1 plus 3-minute secretion during IVGTT.

Insulin resistance was evaluated using a homeostasis model assessment (HOMA) R index calculated from fasting plasma blood glucose and serum insulin concentration according to Matthews and cols. (24).

### Statistical analysis

Statistical analysis was performed using SPSS version 10.0 for Windows, SPSS Inc. A sample size was calculated to detect type 2 errors, taking into account the low prevalence of GADA and IA2A positivity in subjects with ATD. Data are expressed as the mean (SD) or as median and range. Data that were normally distributed were log transformed for analysis. Statistical differences in antibodies indices between groups were tested using ANOVA and Bonferroni. Homogeneity of variances was tested using Levene’s test. Differences in the frequencies of antibodies between HT, GD and controls were tested by chi-Square and Fisher’s test when appropriate. A *p* value < 0.05 was considered statistically significant.

## RESULTS

The clinical features of GD (n = 181), HT (n = 143) and healthy control subjects (n = 93) are represented in Table 1.

**Table 1:** Clinical and laboratory characteristics in ATD patients and control individuals

	ATD		Control
	GD*	HT**	
N (F/M)	181 (151/30)	143 (132/11)	93 (53/40)
Age (y.o)	40 (16-71)	48 (16-77)^a^	28 (18-58)
Time from ATD diagnosis (years)	4 (0.2-35)	4 (0.1-39)	
BMI (kg/m^2^)	26 ± 4	28 ± 6.2	25 ± 3.6
GADA (U/ml)	0.05 (0.0-324.89)	0.14 (0.0-57.85)^b^	0.0 (0.0-1.61)
IA2A (U/ml)	0.02 (0.0-1.46)	0.02 (0.02-1.53)	0.01 (0.01-0.77)
GADA+ (%)	13/181 (7.2)	3/143 (2.0)	0/93 (0.0)
IA2A+ (%)	1/146 (0.7)	1/113 (0)	0/30 (0)
TPOAb (U/ml)	72 (0.1-80)	222 (0.0-3116)	0.8 (0.1-133)

* GD: Graves’ disease. ** HT: Hashimoto’s thyroiditis.

Data are expressed as median (range) or mean ± SD.

^a^ (HT vs GD and C; p < 0.01); ^b^(GD vs HT; p < 0.01).

GADA and IA2A were detected at 4.9% (16/324) and 0.7% (2/259), respectively, in the ATD patients. We did not find any patient with both antibodies. There was no difference between the clinical characteristics of GADA and/or IA2A positive and negative patients [(age 35 (22–68) vs 43 (16–77) years old, BMI (24.1 ± 3.3 and 27.3 ± 5.4 kg/m^2^), such as disease duration 3.3 (0.3–20) and 4.0 (0.1–39) years], respectively.

None of the 93 control group members was positive for GADA or IA2A.

**Figure 1:**
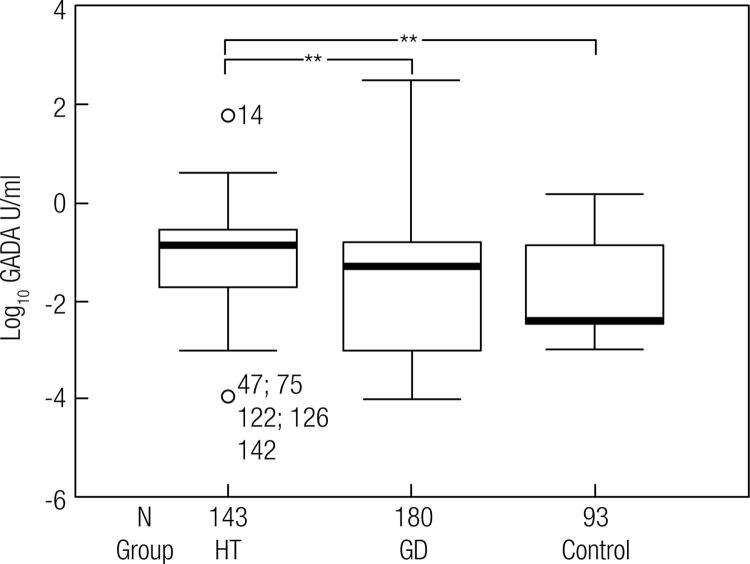
GADA level distribution in the groups. Values transformed to log10. ** p < 0.001 (HT vs GD and controls) obtained by ANOVA.

**Figure 2:**
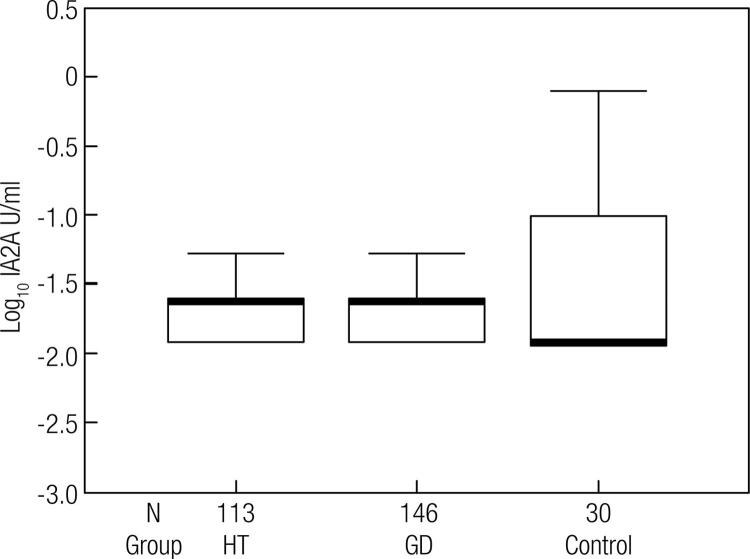
IA2A level distribution in the groups. Values transformed to log10.

Data on GD and HT individualized are shown in Table 1. Apart from the high variability of GADA titers in both ATD, the values in HT were significantly higher than in GD (*p* < 0.001) and controls (*p* < 0.001), but there was no difference between GD and controls (Figures 1 and 2).

In patients with positive pancreatic islet cell antibodies, we also analyzed GADA titers according to the length of time since the clinical diagnosis of ATD. We shared them in 4 groups: ≤ 1 yr., > 1 ≤ 5 yr., > 5 ≤ 10 yr. and > 10 yrs. The GADA titers were 70.7 ± 38.3, 61.2 ± 107.7, 16.5 ± 15.7, 4.7 ± 0.9 U/ml, respectively, for these groups. The median figures of their ages were in the same order: 22 (22–54), 29 (23–56), 43 (36–68), 44 (40–48) years old. Patients with less than five years of ATD seemed to have higher titers of GADA and be younger than the others.

Eleven positive and 20 negative pancreatic islet cell auto-antibodies euthyroid ATD patients with similar ages, genders and BMIs accepted being tested for FPIR, insulin resistance (HOMA-R) and OGTT (Table 2).

**Table 2:** Clinical and laboratorial features of positive (Ab+) and negative (Ab-) pancreatic islet-cell autoantibodies ATD patients analyzed in relation to βcell function and insulin resistance

	Ab+	Ab-	p
Gender (F/M)	8/3	17/3	
BMI (kg/m^2^)	24.3 ± 3.2	24.9 ± 3.2	
GADA (U/ml)	14.2 (0.00-324.9)	0.06 (0.00-0.56)	< 0.001
IA2Ab (U/ml)	0.2 (0.01-1.53)	0.0 (0.01-0.37)	< 0.001
FPIR (pmol/L)	786.2 ± 534.0	984.0 ± 717.6	
FPG (mmol/L)	4.78 ± 0.43	4.72 ± 0.47	
2hPG (mmol/L)	6.07 ± 1.52	5.27 ± 1.31	
HOMA-R	1.09 ± 0.6	1.33 ± 0.9	

Data were expressed as median values at time of the last evaluation.

Overall, there was not a significant difference between positive and negative GADA/IA2A euthyroid ATD patients in relation to first-phase insulin secretion, insulin resistance or oral glucose tolerance.

However, when they were individually analyzed, we saw that 27.2% (3/11) of the GADA/IA2A+ against 5% (1/20) GADA/IA2A- showed low FPIR. Of these GADA/IA2A+/low FPIR, one had GD, with very high GADA titers (324.8 U/ml) and impaired fasting glucose (FPG = 6.83 mmol/L or 123 mg/dl), another had HT with low GADA titers (2.1 U/ml) and was overweight (BMI: 27 kg/m^2^) and the third subject had HT as well. Both with hypothyroidism had normal glucose tolerance. However, the GADA/IA2A-/low FPIR patient had GD and normal glucose tolerance.

## DISCUSSION

In this study, we showed that the prevalence of GAD/IA2 autoantibodies in Brazilian, non-diabetic, euthyroid ATD patients is higher than in the healthy population studied. Despite the similar frequency of GAD and IA2 autoantibodies in patients with HT or GD, GADA titers in HT patients were higher than those observed in GD patients. Nevertheless, GAD/IA2 autoantibodies positivity per se does not seem to be associated with any disturbance in insulin secretion or glucose tolerance in euthyroid ATD patients at the time of this study.

GADA and IA2A frequencies were 4.9% and 0.7%, respectively, in the ATD patients. These frequencies were higher than those found previously in our healthy population (0.5% and 0.0%) (22) and demonstrated in similar patients in other studies (22-24). However, when the frequency of GADA in GD was compared to HT, the former (7.2%) tended to be slightly higher than the latter (2%). These data confirm our previous study when we found a higher prevalence of GADA in GD in a small group of patients with the same age and anthropometric clinical characteristics (19).

In Swedish patients, GADA was detected in 13% of newly diagnosed GD patients without clinical diabetes mellitus and in 3.4% of HT patients (18,25). In Japanese patients, GADA was found in 6% of GD and 7.9% of HT patients (17) although the time since thyroid disease diagnosis was not reported, and the patients were in different degrees of thyroid function. In our patients, the median time of thyroid disease clinical diagnosis was 4 years, with some of them with more than 30 years.

We observed that when ATD were pancreatic islet-cell Ab-positive patients, they seemed to have thyroid disease for a shorter time and were younger, although the sample was too small to test this hypothesis statistically.

We found higher GADA titers in HT than GD patients despite the same age and thyroid-disease duration in both groups of subjects. The presence of higher GADA at the time of diagnosis in organ-specific autoimmune diseases other than T1AD can be explained by the higher influence of an antigenic environment. Furthermore, it has been reported that the GAD65 protein is found in the brain and even in lower concentrations in the thyroid, pituitary, kidney, liver, adrenal, ovary and testes in addition to pancreatic islets, raising the possibility of triggering antibody production based on the polyclonal B-lymphocyte production in ATD patients (17). In fact, subjects with ATD also show a high prevalence of autoantibodies against non-thyroid specific antigens (26).

The relevance of GADA to T1AD development must be considered one step of the whole process, such as genes conferring susceptibility to or protecting from T1AD, the presence of others’ pancreatic antibodies, their titer levels, their persistence and the autoantibody epitope specificity. The autoimmune disease is the final phase of a process starting with auto recognition, passing through immunity with the appearance of autoantibodies and finally leading to cell destruction and autoimmune disease (27).

A study in a group of Japanese patients who had been diagnosed initially with non-insulin dependent diabetes mellitus (NIDDM) showed that those with higher GADA titers progressed rapidly to an insulin-dependent state (28-30). However, in a study of stratification of diabetes risk in T1AD’s first-degree relatives, it was found that high titers of IA2A and IAA, though not GADA, were associated with an increased risk for clinical diabetes (29).

A study demonstrated that only the simultaneous presence of GADA and “classical” islet cell antibody (ICA) was associated with a loss of beta-cell function during follow-up and an increased risk of progression toward insulin dependency and low BMI (30).

We found that ATD patients with positive pancreatic islet cell Ab presented a downward tendency in the FPIR results besides normal FPG and glucose tolerance.

It has been shown that positive GADA HT patients had similar FPG and insulin concentrations than negative GADA subjects; however, when submitted to arginine injection, they showed a decline in the insulin secretory capacity and the glucagon response to arginine (25).

Insulin resistance in patients with islet autoimmunity could have a genetic-constitutional basis and might lead to the earlier appearance of hyperglycemia when beta-cell function is compromised by autoimmune disease (31). In a recent study, this research group showed that T1AD relatives that were positive for islet antibodies and progressed most rapidly to diabetes had shown a subtle disturbance of insulin-glucose homeostasis years before the onset of symptoms, distinguished by greater insulin resistance for their level of insulin secretion. The degree of insulin sensitivity estimated by HR was similar between positive and negative GADA/IA2A groups and as expected, showed a positive relation with BMI (rS = 0.52; *p* = 0.0034).

This study was limited by a small sample of GADA/IA2A-positive AITD patients in relation to FPIR and glucose tolerance and by the short follow-up of these patients.

In summary, the prevalence of GADA/IA2A was higher in non-diabetic ATD patients than in healthy controls. GADA prevalence was similar in HT and GD, but higher titers of these autoantibodies were found in the first. The degree of insulin resistance and glucose tolerance was similar between positive and negative GADA/IA2A euthyroid non-diabetic ATD patients. Moreover, we found only a tendency for low FPIR in GADA/IA2A-positive individuals. This may correspond to subclinical insulitis.
